# A four specimen-pooling scheme reliably detects SARS-CoV-2 and influenza viruses using the BioFire FilmArray Respiratory Panel 2.1

**DOI:** 10.1038/s41598-022-09039-6

**Published:** 2022-03-23

**Authors:** Charlene Ranadheera, Greg J. German, Laura Steven, Dale Eung, Dmytro Lyubashenko, Jessica C. Pepin, Marko Zivcec, Kym Antonation, Cindi R. Corbett

**Affiliations:** 1 Health Security and Response Division, National Microbiology Laboratory, Public Health Agency of Canada, Winnipeg, MB Canada; 2 Queen Elizabeth Hospital, Charlottetown, PE Canada; 3 Department of Laboratory Medicine and Pathology, University of Toronto, Toronto, ON Canada; 4 Stanton Territorial Hospital, Yellowknife, NT Canada; 5 Department of Medical Microbiology and Infectious Diseases, University of Manitoba, Winnipeg, MB Canada

**Keywords:** Microbiology, Molecular biology, Diagnosis

## Abstract

The COVID-19 pandemic required increased testing capacity, enabling rapid case identification and effective contract tracing to reduce transmission of disease. The BioFire FilmArray is a fully automated nucleic acid amplification test system providing specificity and sensitivity associated with gold standard molecular methods. The FilmArray Respiratory Panel 2.1 targets 22 viral and bacterial pathogens, including SARS-CoV-2 and influenza virus. While each panel provides a robust output of information regarding pathogen detection, the specimen throughput is low. This study evaluates the FilmArray Respiratory Panel 2.1 using 33 pools of contrived nasal samples and 22 pools of clinical nasopharyngeal specimens to determine the feasibility of increasing testing capacity, while maintaining detection of both SARS-CoV-2 and influenza virus. We observed 100% detection and 90% positive agreement for SARS-CoV-2 and 98% detection and 95% positive agreement for influenza viruses with pools of contrived or clinical specimens, respectively. While discordant results were mainly attributed to loss in sensitivity, the sensitivity of the pooling assay was well within accepted limits of detection for a nucleic acid amplification test. Overall, this study provides evidence supporting the use of pooling patient specimens, one in four with the FilmArray Respiratory Panel 2.1 for the detection of SARS-CoV-2 and influenza virus.

## Introduction

A novel coronavirus, later termed severe acute respiratory syndrome coronavirus 2 (SARS-CoV-2), emerged in late December 2019, when reports of several cases of pneumonia of unknown origin were reported from China. On March 11, 2020, the World Health Organization declared a Coronavirus Disease 2019 (COVID-19) pandemic and as of February 20, 2022, there have been over 422 million cases and 5.9 million reported deaths worldwide^1^. The surge of cases worldwide has made it necessary for countries to increase their testing capacity, by equipping new facilities with appropriate equipment, test reagents, and training staff on appropriate handling measures.

Historically, in Canada, remote and isolated communities experience delayed access to test results attributed to specimen transport time to urban centres. Increasing testing demands compounded this issue across all urban centres during the pandemic. To alleviate this burden, the Public Health Agency of Canada supported a Northern, Remote, and Isolated Communities Initiative, as recommended by the Canadian Public Health Laboratory Network, to support building capacity in remote laboratories through provision of testing supplies and training. This initiative focused on the distribution of multiple different platforms, which included the BioFire FilmArray diagnostic system as ways to support increased testing capacity.

The BioFire FilmArray diagnostic system provides users with a rapid, easy to use testing approach, allowing laboratory workers that may not normally be able to conduct nucleic acid amplification tests (NAAT), to do so in a safer manner. It is a fully automated system coupling a nucleic acid extraction and purification process with a multiplexed reverse-transcription nested PCR reaction. Using a nasopharyngeal swab sample in 3 ml of transport media, the FilmArray Respiratory Panel (RP) 2.1 targets 22 viral and bacterial pathogens known to cause respiratory illness, including SARS-CoV-2 and influenza viruses. The FilmArray RP2.1 builds upon the former FilmArray RP2 with the addition of a SARS-CoV-2 test. A study by Leber et al., evaluating the FilmArray RP2 panel was done by analyzing 1612 clinical samples, subsequently generating 33,843 test results, and demonstrated acceptable positive and negative percent agreements, 99.2% and 99.3%, respectively^2^. The SARS-CoV-2 assay targets two regions within the S and M genes, has a limit of detection (LOD) of approximately 160 copies/ml, and a lack of cross-reactivity with other pathogens^3^. The highly conserved FilmArray RP2.1 SARS-CoV-2 targets are able to detect the recently emerged variants of concern, including B.1.1.7 (Alpha), B.1.351 (Beta), B1.617.2 (Delta), P.1 (Gamma), and Omicron B.1.1.529^4^.

Throughout the COVID-19 pandemic, every country experienced increased testing demands and global shortages of sampling tools and diagnostic reagents were observed. Sample pooling was a relevant method to increase testing capacity while conserving reagents and reducing economic strain. Many studies have demonstrated that the potential sensitivity loss of molecular assays when pooling patient samples using traditional real-time qPCR methods for the detection of SARS-CoV-2 does not impact positive agreement, and thus patient diagnosis^5–11^. In this study, we evaluated the potential for pooling multiple specimens using the FilmArray RP2.1 to increase testing capacity and laboratory turn around times. In an effort to test the FilmArray RP2.1 under the most stringent conditions, sample pools were made using clinical or contrived-clinical samples containing one SARS-CoV-2 sample and three samples each comprised of at least one challenge pathogen, including human coronaviruses, influenza viruses, respiratory syncytial virus (RSV) and rhinoviruses (RV), at various viral loads.

## Results

### Limit of detection after pooling contrived-clinical samples

**Figure 1:**
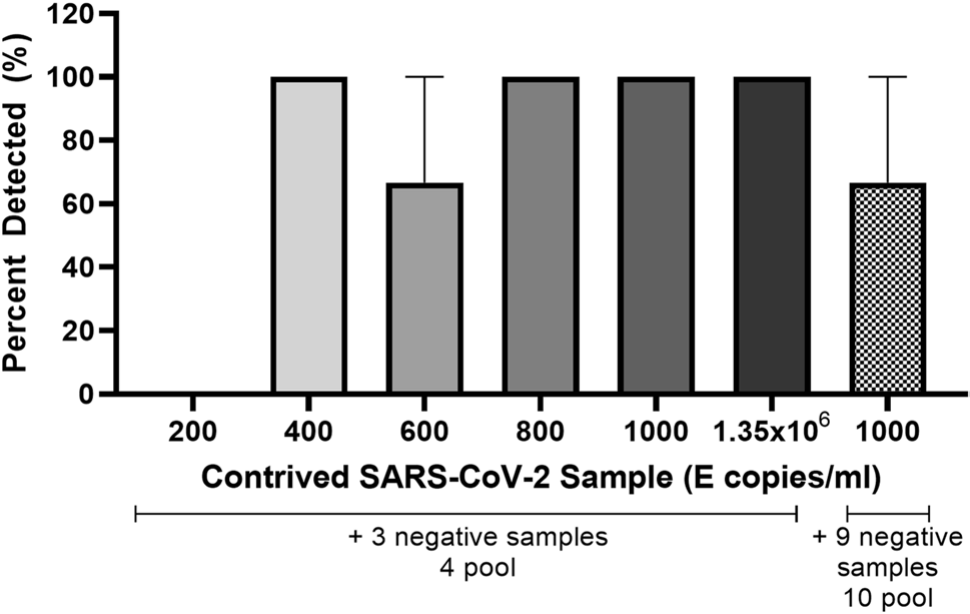
Sensitivity of the FilmArray RP2.1 for the detection of SARS-CoV-2 after specimen pooling. One contrived sample containing, 200, 400, 600, 800 or 1000 E copies/ml, was equally mixed with three negative contrived samples, with the exception of the last sample, which was equally mixed with nine negative contrived samples. The FilmArray RP2.1 was used to detect the presence of SARS-CoV-2. Each bar denotes a separate pool. Each pool was tested in triplicate and the mean and standard error are presented.

Four and ten sample-pools, made from quantified irradiated SARS-CoV-2 diluted in pooled, negative deep nasal swabs in viral transport media, were tested for the ability to detect the presence of SARS-CoV-2. The LOD with 100% accuracy for the FilmArray RP2.1 is 160 copies/ml^3^. Similarly, the FilmArray RP2.1 reliably detected SARS-CoV-2 samples containing 800 E gene copies/ml that were pooled with three negative samples, corresponding to a final concentration of 200 copies/ml (Fig. 1). At lower concentrations, detection was variable; a sample containing 600 copies/ml (or 150 copies/ml after pooling) had a 67% detection rate, while a sample containing 400 copies/ml (or 100 copies/ml after pooling) was consistently detected (Fig. 1). Using a 10 sample-pooling strategy the LOD was greater than 1000 copies/ml when pooled with nine negative samples (Fig. 1). The 10 sample-pooling strategy was not investigated further due to increased signal loss: this study will focus on a four sample-pooling logic.

**Figure 2:**
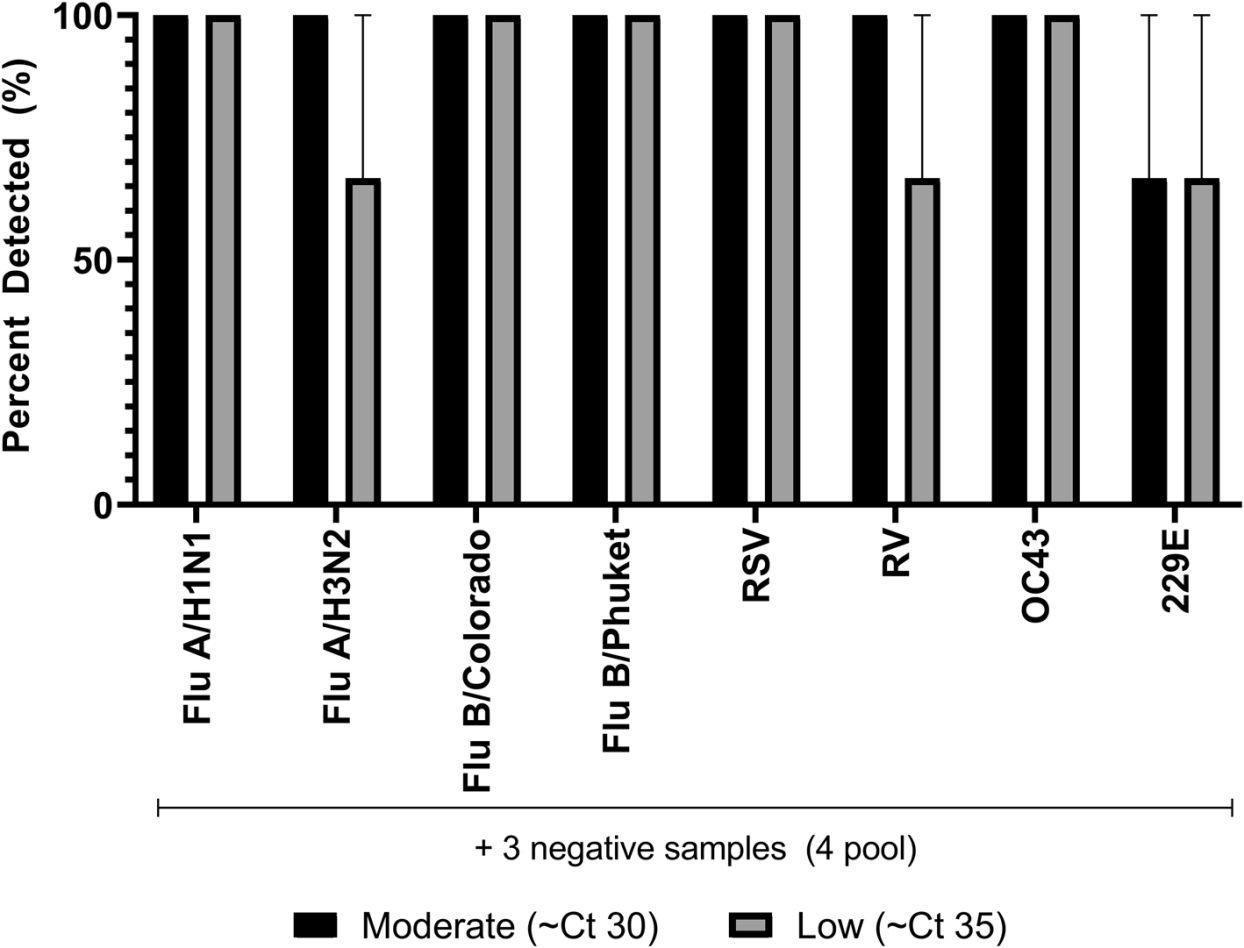
Detection of challenge respiratory viruses using the FilmArray RP2.1 after specimen pooling. A contrived-clinical sample containing a low (~ Ct 35) or moderate (~ Ct 30) concentration of either Flu A, FluB, RSV, RV, OC43, and 229E was equally mixed with three negative contrived-clinical samples. Each pool was tested in triplicate and the mean and standard error are presented.

In a similar design, various irradiated respiratory viruses were diluted in a negative background matrix so that each virus had representative low and moderate sample concentrations. Each sample was pooled with three negative contrived-clinical samples. The FilmArray RP2.1 reliably detected FluA/H1N1, FluB/Colorado, FluB/Phuket, RSV, and OC43 at low concentrations of virus (~ Ct 35), while FluA/H3N2, RV and 229E had 67% detection rate at this concentration (Fig. 2). Using the FilmArray RP2.1, moderate virus concentrations (~ Ct 30) had a 100% detection rate for all respiratory viruses except for 229E, which had a 67% detection rate (Fig. 2).

### Performance of the FilmArray RP2.1 after pooling contrived-clinical samples

**Figure 3:**
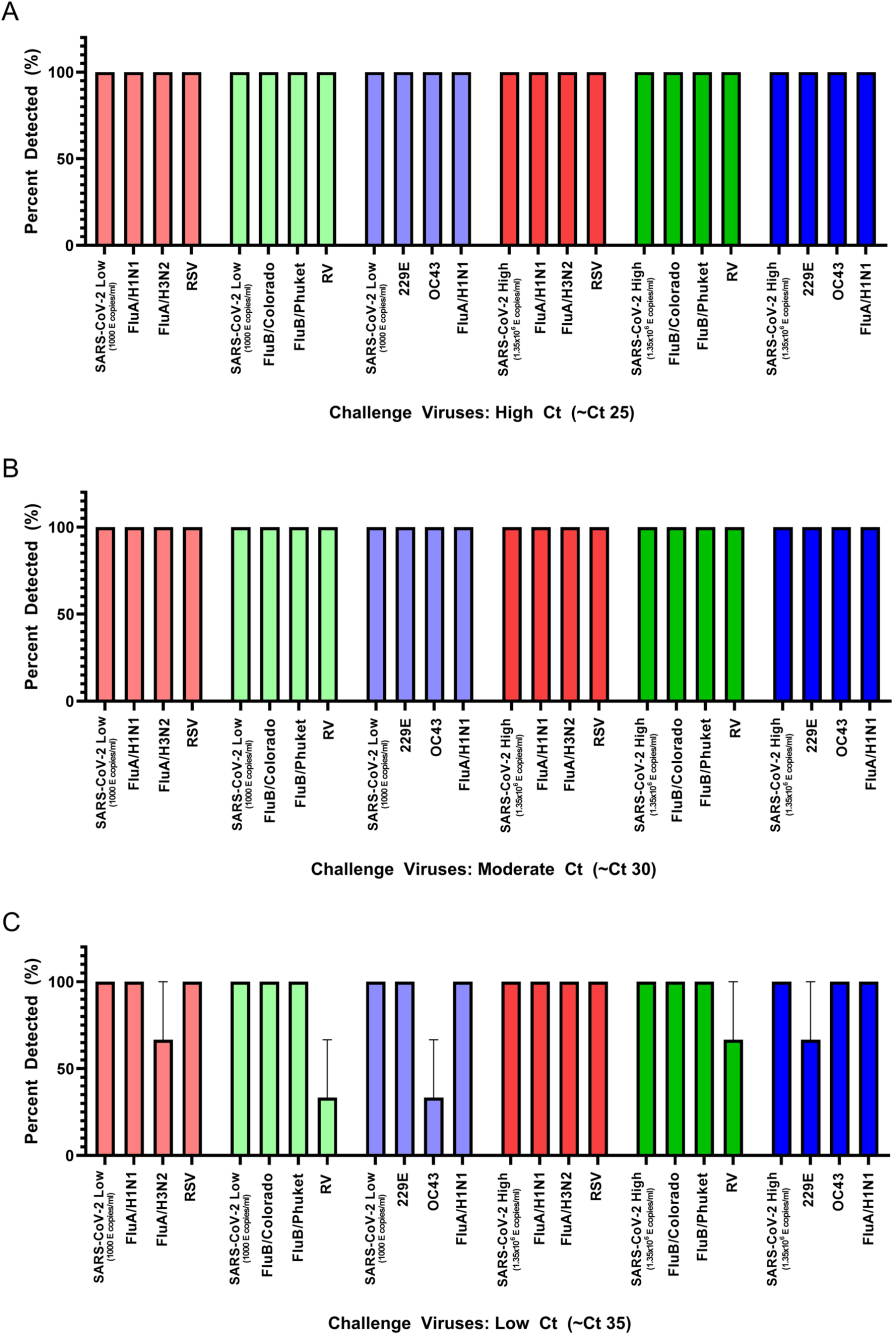
Detection of SARS-CoV-2 and challenge respiratory viruses after a four specimen-pooling strategy using the FilmArray RP2.1. A contrived-clinical sample containing 1000 E copies/ml or 1.35 × 10^6^ E copies/ml of SARS-CoV-2 was equally mixed with three contrived samples containing (**A**) high (~ Ct 25), (**B**) moderate (~ Ct 30), or (**C**) low (~ Ct 35) amounts of either Flu A, FluB, RSV, RV, OC43, and 229E. The FilmArray RP2.1 was employed to detect the presence of SARS-CoV-2. Each coloured set denotes a separate pool. Each pool was tested in triplicate and the mean and standard error are presented.

In the presence of high concentrations of challenge viruses, the FilmArray RP2.1 was able to detect SARS-CoV-2 at both 1000 and 1.35 × 10^6^ E gene copies/ml of SARS-CoV-2 (Fig. 3A). Additionally, this assay was also able to detect the presence of respective viruses in each pool consistently (Fig. 3A). From 18 reactions, there were no discordant results recorded. Likewise, similar observations were made when SARS-CoV-2 samples were pooled with samples containing moderate concentrations of challenge viruses (Fig. 3B). When SARS-CoV-2 samples containing 1000 or 1.35 × 10^6^ copies/ml of SARS-CoV-2 were combined with samples containing low concentrations of challenge viruses, discordant results were observed. While SARS-CoV-2 was detected effectively in each pool, the identification of the challenge viruses was variable (Fig. 3C). The first pool missed identifying FluA/H3N2 in 1/3 replicates, the second and fifth pools missed detecting RV in 67% and 33% of replicates, respectively, and the sixth pool failed to identify 229E in 1/3 replicates (Fig. 3C). Together with the data obtained in Fig. 2, these results support the finding that pooling low concentration samples containing these viruses impacted detection due to a dilution below the viral target’s LOD. Interestingly, while OC43 was detected effectively at the high Ct benchmark test by itself (Fig. 2), it was not reliably identified in the third pool, where it was mixed with other challenge viruses. At this same benchmark Ct level, the OC43 target went undetected 67% of the time (Fig. 3C). Overall, the four sample-pooling logic effectively preserved detection of all 54 contrived-clinical samples containing SARS-CoV-2. Additionally, 114 influenza virus positive samples were included and detected 98% of the time, indicating its potential for dual testing.

### Performance of the FilmArray RP2.1 after pooling clinical specimens

Fifty-six clinical samples, positive for the presence of Adenovirus, Flu A, Flu B, HKU1, NL63, RSV, RV and SARS-CoV-2, were collected between December 2019 and March 2021, and pooled together in various combinations, generating 22 pools. Each pool was tested in triplicate. Thirteen pools (Pools 1–13) contained one SARS-CoV-2 sample and three samples with at least one challenge virus present (Supplemental Table S1). The remaining nine pools (Pools 14–22) consisted of four samples that only contained challenge viruses (Supplemental Table S1). SARS-CoV-2 was identified consistently from all pools except Pool 2 and Pool 5, resulting in a 90% positive agreement (Table 1). SARS-CoV-2 was detected in 2/3 replicates from Pool 5 and was missed in all replicates from Pool 2 (Supplemental Table S1). SARS-CoV-2 was not detected in Pools 14–22, demonstrating a 100% negative agreement (Table 1, Supplemental Table S1). Flu A and Flu B had positive agreements of 98% and 92%, respectively, while Adenovirus and RV had positive agreements of 67% and 91%, respectively (Table 1). HKU1, NL63, and RSV all demonstrated 100% positive agreement (Table 1). All of the targets investigated here demonstrated a 100% negative agreement for all but one pool. Unexpectedly, PIV-1 was detected in 1/3 replicates in pool 5, however, there was no detectable presence of PIV-1 in those samples when individually tested by qRT-PCR (Table 1, Supplemental Table S1). Overall, SARS-CoV-2 and influenza virus detection was not significantly impaired when a four specimen-pooling strategy was employed; however, an expected minor loss in sensitivity was observed.

**Table 1:** Performance of the FilmArray RP2.1 Test panel for each pathogen target from pooled clinical samples.

Target pathogen	Total detected by the FilmArray RP2.1	Total expected positive	Positive percent agreement (%)	Total not detected by the FilmArray RP2.1	Total expected negative	Negative percent agreement (%)
Adenovirus	2	3	67	63	63	100
Flu A	50	51	98	15	15	100
Flu B	44	48	92	18	18	100
HKU1	9	9	100	54	54	100
NL63	9	9	100	57	57	100
PIV-1	0	0	n/a	65	66	98
RSV	60	60	100	6	6	100
RV	60	66	91	0	0	n/a
SARS-CoV-2	35	39	90	27	27	100
Remaining targets	0	0	n/a	66	66	100

## Discussion

Implementing pooling schemes using rapid, automated systems has been successful when detecting a single target. For example, the Cepheid Xpert Xpress SARS-CoV-2 assay was effective when implementing a four or six specimen-pooling logic^12,13^. While the authors assessed a small number of pools, 13 and 14 pools respectively, all results indicated 100% positive agreement and 100% negative agreement for SARS-CoV-2^12,13^. Similar results were observed by Anderson et al. utilizing the FilmArray COVID-19 test with a ten specimen-pooling logic^14^. One limitation to the aforementioned studies was the lack of low titer specimens used^13,14^. Sample populations mainly consisted of specimens with Ct values between 20 and 33, therefore, not fully assessing the dynamic range of the assay and the ability to detect a sample containing low amounts of SARS-CoV-2^13,14^. Likewise, pooling influenza virus specimens is common practice, Canadian laboratories routinely use a four specimen-pooling logic; while others use a ten-specimen pooling scheme^15,16^. There has been hesitation to pool specimens with the FilmArray RP2.1 due to multiple targets being tested simultaneously. While each panel tests for 22 different pathogens concurrently, each individual gene target is assayed independently on a multi-well array, distinguishing it from typical single-reaction multiplexed qPCR systems; thereby alleviating concerns normally associated with multiplexed assays, for example sensitivity losses and increasing false positive and negative results due to primer dimers and inappropriate cross hybridization. Therefore, the ability to pool patient samples using the FilmArray RP2.1 test panel could have significant value.

Considerations should be made to determine the benefits of specimen pooling. First, retesting of individual specimens are required from a positive pool; therefore, the community-based pathogen incidence rate should be considered to determine if pooling specimens would be of value or hindrance. Pooling specimens to screen for SARS-CoV-2 outbreak investigations when low-incident rates are expected; thereby increasing testing capacity upwards of fourfold and reducing turn around times, which may be useful when expedited results are needed. It should be noted that FilmArray RP2.1 testing on asymptomatic patients would have to be further assessed, since this has not been evaluated by the manufacturer^3^. It has been suggested that a case positivity rate ranging from 0 to 7% would be beneficial; reducing testing outputs by approximately 57%, when a four-specimen pooling strategy is employed^5,17^. This would be highly beneficial for northern and remote centres in Canada, where prior to the emergence of the B.1.1.529 (omicron) VOC, the SARS-CoV-2 positivity rate was consistently below 5% since the start of the pandemic^18^. Concurrently, the incidence of influenza virus in these locations has been below 1%^19^. In such a situation, the estimated 70% reduction in expenses would be fortuitous due the costly nature of the FilmArray RP2.1 test kits. Accordingly, remote and northern sites in Canada have successfully implemented pooling strategies during times of low incidence of SARS-CoV-2 to increase testing outputs, citing reduced financial burden, turn around times and staffing requirements. Implementing the FilmArray RP2.1 test kit, pooling of specimens during low incidence of SARS-CoV-2 and/or influenza viruses would be recommended for symptomatic adult populations, while children would be tested individually as there would be additional value to gain insights for multiple targets, including detection of RSV. With the emergence of B.1.1.529 (Omicron), the incident rate in these remote locations in Canada have increased but most regions continue to range between 4–8%^18^, while influenza virus rates continue to remain below 1%^19^, indicating that pooling samples would remain fortuitous in these locations. However, in certain situations, COVID-19 incidence has increased to 17%^18^, in which case the need for repeat testing will increase causing excessive reagent use and turn around times. As such, specimen pooling during times of high incidence should be avoided. Similar to the approach taken for pooling influenza specimens in Canada^16^, we chose to implement a four-specimen pooling strategy to ensure adequate sensitivity is retained. In our hands, we determined that a single sample would need to contain 800 SARS-CoV-2 E gene copies/ml to ensure 100% detection in a four specimen-pooling scenario, compared to 160 copies/ml when individually testing samples. This increased LOD is still within acceptable limits, since other widely used diagnostic assays have lower sensitivities. For example, the manufacturer states that the Applied Biosystems TaqPath COVID-19 Combo Kit has a LOD of approximately 2000 copies/ml^20^. Therefore, it was deemed reasonable that a four-specimen pooling strategy using the FilmArray RP2.1 was appropriate and the slight loss of sensitivity was acceptable. While the scope of this study is focused on detecting the presence of SARS-CoV-2 and influenza viruses, a reduction in detection of RV, 229E and OC43 after pooling was observed.

Although a minor loss in sensitivity was recognized, the presence of competing viral pathogens did not negatively affect the ability of the FilmArray RP2.1 to identify the presence of SARS-CoV-2 and influenza viruses. More specifically, heavily challenged pools comprised of one SARS-CoV-2 sample and three samples containing three to four competing pathogens were still detected at low concentrations of SARS-CoV-2 and influenza virus. Pooled contrived-clinical samples elicited 100% detection and 90% positive/100% negative agreement in pooled clinical samples for SARS-CoV-2. The latter decrease in positive percent agreement are not surprising, since one sample had very low levels of SARS-CoV-2 present (Ct 38.4) and subsequent pooling likely diluted the specimen below the LOD for the assay (800 E copies/ml/specimen). However, the inability to detect SARS-CoV-2 in 1 out of 3 replicates in Pool 5 was surprising since the specimen had a moderate amount of viral RNA present (Ct 30.6). One could speculate this outcome was due to a technical error rather than a loss of sensitivity, such as insufficient mixing before and after addition to the sample buffer. Pooled contrived-clinical samples elicited 98% detection and 95% positive/100% negative agreement in pooled clinical samples for influenza virus. It is not surprising that there was a loss in Flu A and B detection, since all but one discordant result had original Ct values > 39; one Flu B discordant result with moderate viral RNA levels (Ct 32.9) remains unexplained, except for the possibility of a technical error. The discordant results obtained for the other challenge pathogens were likely caused by dilution of the sample below the gene target’s LOD, while one discordant result where one out of three replicates was positive for PIV-1 remains unexplained. A previous study evaluating the progenitor FilmArray RP2 similarly described false positive results, which could not be reproduced by traditional qPCR-based assays; however, it notes that influenza virus targets were detected reliably^2^.

Overall, this study assessing 55 clinical- and contrived clinical sample pools, demonstrates how a four specimen-pooling logic using the FilmArray RP2.1 would be a suitable strategy for the detection of SARS-CoV-2 samples during a time of low to moderate incidence; subsequently increasing testing capacity and reducing turn around times. Additionally, influenza viruses were reliably detected throughout this study, indicating that both SARS-CoV-2 and influenza virus testing could be done simultaneously on symptomatic adults with a four specimen-pooling strategy using the FilmArray RP2.1, thereby providing another potential avenue to streamline workflow in remote community hospital laboratories. Timely provision of test results is an essential component to public health precautions, as it allows for more rapid containment and a decreased risk for further transmission.

## Materials and methods

### Virus propagation

Influenza viruses, A/Michigan/45/2015 (Flu A/H1N1), A/Kansas/14/2017 (Flu A/H3N2), B/Phuket/3073/2013 (Flu B/Phuket), and B/Colorado/06/2017 (FluB/Colorado), were propagated in MDCK cells using EMEM supplemented with 0.1% bovine serum albumin (Hyclone; Logan, UT, USA), 1% l-glutamine, 1 mM Sodium Pyruvate, 1% non-essential amino acids, 1 × penicillin–streptomycin, 25 mM HEPES (ThermoFisher Scientific, Waltham, MA USA), 1% l-glutamine, and 1 mg/ml TPCK-treated trypsin (Millipore Sigma; Oakville, ON, Canada). SARS-CoV-2 (hCoV-19/Canada/ON_ON-VIDO-01-2/2020; Accession ID: EPI_ISL_425177), human Rhinovirus 49 (Strain 8213, ATCC VR-1644), and RSV (Strain long, ATCC VR-26) were amplified in Vero cells, HeLa cells, or Hep2 cells, respectively, using EMEM supplemented with 2% FBS, 1 mM Sodium Pyruvate, 1% non-essential amino acids, 1 × penicillin–streptomycin, and 1% l-glutamine. Human Coronavirus 229E (229E, ATCC VR-740) and Human Coronavirus OC43 (OC43, ATCC VR-1558) were amplified in MRC7 cells using DMEM supplemented with 2% FBS, 1 mM Sodium Pyruvate, 1% non-essential amino acids, 1 × penicillin–streptomycin, and 1% l-glutamine. Virus stocks were inactivated by gamma irradiation; SARS-CoV-2 was irradiated using 3Mrads and the remaining viruses were irradiated with 2Mrads. All viruses were safety tested prior to use in containment level 2.

### Reverse transcription real-time PCR

Nucleic acids were extracted from 265 µl of nasopharyngeal swab medium using the MagMax Viral RNA Isolation Kit on the KingFisher Flex (Fisher Scientific; Ottawa, ON, Canada) according to manufacturer instructions. Five microliters of nucleic acid was added to a 20 µl reaction using QuantiNova RT-PCR kit (Qiagen; Toronto, ON, Canada), as per manufacturers instructions, on the QuantStudio 5 (Fisher Scientific; Ottawa, ON, Canada). The primers and probes used were previously described for the detection of RV^21^, Flu A, Flu B and RSV^22^, Parainfluenza virus 1 (PIV-1)^23^, and SARS-CoV-2^24^. The following primers were developed in-house to detect the presence of human coronaviruses:

- NL63: 5′-GTGGTGTACTTCTTGTTGATGG, 3′-GGTACTAGGCGTAGCAACTATT, 56-FAM-AGGCAACTG-ZEN-ACCCACTTGAACACG-3IABkFQ;
- OC43: 5′-GGACCTCATTTGTGTGCAAAGTT, 3′-GCAGCCAAAGTGTATCCACTGA, JUN-TCAAAGTGTTGCCTCCACTGCTCTCAGA-QSY;
- 229E: 5′-CACTTTTGACAAGAAAGCGTTTACAC, 3′-CTGCGACCAGCCACTCTACTAC, VIC-TAGCAGCGTCATACCTAGCTTGCCCACA-QSY; and
- HKU1: 5′-CTGGTTGGTGTTGGTGAACATT, 3′-TGATCCATCCAATACACCACACT, FAM-TGCAGGGTTCGGTGTT-MGBNFQ.

### Negative background matrix

Healthy volunteers provided a nasal swab specimen. Each nostril was swabbed 10 times daily and collected into 100 ml of viral transport media (Corning; Corning, NY, USA). The nasal swab/viral transport media solution from all participants were pooled together to anonymise. The pooled negative background matrix was screened using the FilmArray RP2.1 to verify absence of 22 respiratory pathogens.

### Contrived-clinical samples

Contrived-clinical samples containing 200, 400, 600, 800 and 1000 copies/ml of the SARS-CoV-2 E gene were generated by diluting quantified amounts of irradiated SARS-CoV-2 into the pooled negative background matrix. These samples were combined with three or nine negative, contrived-clinical samples and were tested using the FilmArray RP2.1.

Irradiated virus was added to negative background matrix. Contrived-clinical SARS-CoV-2 samples containing either 1000 or 1.35 × 10^6^ E copies/ml were generated, while other irradiated respiratory viruses were diluted to low (~ Ct 35), moderate (~ Ct 30) and high (~ Ct 25) titer specimens. Contrived-clinical samples containing SARS-CoV-2 were combined with three contrived-clinical samples containing other respiratory viruses and run on the FilmArray RP2.1.

### Clinical specimens

Fifty-six archived nasopharyngeal swab samples were collected across Canada between December 2019 and March 2021. Pathogens identified in these clinical specimens included Adenovirus, Flu A, Flu B, human coronaviruses HKU1 (HKU1) and NL63 (NL63), RV, RSV and SARS-CoV-2. The Applied Biosystems TaqPath COVID-19 Combo Kit (Thermo Fisher Scientific; Ottawa, ON, Canada) was employed as per manufacturer’s instructions for the detection of SARS-CoV-2, while the challenge pathogens were confirmed using the assays previously described. Anonymized clinical samples were pooled and tested in triplicate in various combinations using the FilmArray RP2.1.

## Supplementary Information

Supplementary Table S1.

## Data Availability

The data that support the findings of this study are available on request from the corresponding author, CR.
