# Supplementary material for: A four specimen-pooling scheme reliably detects SARS-CoV-2 and influenza viruses using the BioFire FilmArray Respiratory Panel 2.1
Source: Sci Rep. 2022 Mar 23;12:4947. doi: 10.1038/s41598-022-09039-6 (PMC8942994; doi:10.1038/s41598-022-09039-6)
Supplement: Supplementary file 1 — Supplementary Table S1. [file 41598_2022_9039_MOESM1_ESM.docx]

**Supplementary Table S1: Performance of the FilmArray RP2.1 Test panel using pooled clinical samples**

| ***Pool Number*** | ***Clinical Sample ID*** | ***Target Present*** | | ***Biofire Film Array RP2.1 Detection (N=3)*** | | ***Discordant Results*** | |
| --- | --- | --- | --- | --- | --- | --- | --- |
|  |  | ***Pathogen*** | ***Ct Value*** | ***Pathogen*** | ***Detected (%)*** | ***Pathogen*** | ***Not Detected (%)*** |
| 1 | Sample 31 | RSV  RV | 36.3  27.4 | Adenovirus  Influenza  RV  RSV  SARS-CoV-2 | 67  100  100  100  100 | Adenovirus | 33 |
|  | Sample 2 | Adenovirus  Flu A | n/d  26.5 |  |  |  |  |
|  | Sample 20 | RV | 18.2 |  |  |  |  |
|  | Sample 40 | SARS-CoV-2 | 32.8 |  |  |  |  |
| 2 | Sample 9 | Flu B  NL63 | 25.8  37.4 | Flu B  NL63  RV  RSV  SARS-CoV-2 | 100  100  100  100  0 | SARS-CoV-2 | 100 |
|  | Sample 22 | RV | 20.2 |  |  |  |  |
|  | Sample 28 | RSV | 26.9 |  |  |  |  |
|  | Sample 39 | SARS-CoV-2 | 38.4 |  |  |  |  |
| 3 | Sample 21 | RV | 35.0 | Flu B  RV  RSV  SARS-CoV-2 | 67  0  100  100 | Flu B  RV | 33  100 |
|  | Sample 10 | Flu B | 32.9 |  |  |  |  |
|  | Sample 29 | RSV | 24.7 |  |  |  |  |
|  | Sample 45 | SARS-CoV-2 | 33.0 |  |  |  |  |
| 4 | Sample 6 | Flu A | 39.8 | Flu A  RV  RSV  SARS-CoV-2 | 67  100  100  100 | Flu A | 33 |
|  | Sample 18 | RV | 23.5 |  |  |  |  |
|  | Sample 35 | RSV | 29.6 |  |  |  |  |
|  | Sample 46 | SARS-CoV-2 | 28.8 |  |  |  |  |
| 5 | Sample 8 | Flu A | 22.3 | Flu A  RV  RSV  SARS-CoV-2 | 100  100  100  67 | SARS-CoV-2  PIV-1 | 33  33 |
|  | Sample 17 | RV | 22.4 |  |  |  |  |
|  | Sample 32 | RSV | 39.8 |  |  |  |  |
|  | Sample 47 | SARS-CoV-2 | 30.6 |  |  |  |  |
| 6 | Sample 32 | RSV | 39.8 | Flu A  RV  RSV  SARS-CoV-2 | 100  100  100  100 | - | - |
|  | Sample 7 | Flu A | 27.6 |  |  |  |  |
|  | Sample 27 | RV | 36.1 |  |  |  |  |
|  | Sample 37 | SARS-CoV-2 | 23.8 |  |  |  |  |
| 7 | Sample 5 | Flu A | 26.9 | Flu A  Flu B  RV  SARS-CoV-2 | 100  100  100  100 | - | - |
|  | Sample 49 | Flu B | 31.2 |  |  |  |  |
|  | Sample 23 | RV | 25.4 |  |  |  |  |
|  | Sample 36 | SARS-CoV-2 | 29.4 |  |  |  |  |
| 8 | Sample 19 | RV | 31.7 | Flu B  RV  RSV  SARS-CoV-2 | 100  100  100  100 | - | - |
|  | Sample 11 | Flu B | 24.6 |  |  |  |  |
|  | Sample 33 | RSV | 28.3 |  |  |  |  |
|  | Sample 41 | SARS-CoV-2 | 26.9 |  |  |  |  |
| 9 | Sample 13 | Flu B  HKU1 | 28.8  37.8 | HKU1  Flu B  RV  RSV  SARS-CoV-2 | 100  100  100  100  100 | - | - |
|  | Sample 16 | RV | 28.1 |  |  |  |  |
|  | Sample 34 | RSV | 37.6 |  |  |  |  |
|  | Sample 43 | SARS-CoV-2 | 21.2 |  |  |  |  |
| 10 | Sample 12 | Flu B | 41.7 | Flu B  RV  RSV  SARS-CoV-2 | 0  0  100  100 | Flu B  RV | 100%  100% |
|  | Sample 15 | RV | 31.7 |  |  |  |  |
|  | Sample 34 | RSV | 37.6 |  |  |  |  |
|  | Sample 42 | SARS-CoV-2 | 15.7 |  |  |  |  |
| 11 | Sample 3 | Flu A | 28.8 | Flu A  RV  RSV  SARS-CoV-2 | 100  100  100  100 | - | - |
|  | Sample 14 | RV | 28.7 |  |  |  |  |
|  | Sample 26 | RSV | 32.0 |  |  |  |  |
|  | Sample 44 | SARS-CoV-2 | 18.6 |  |  |  |  |
| 12 | Sample 4 | Flu A | 33.0 | Flu A  Flu B  RV  SARS-CoV-2 | 100  100  100  100 | - | - |
|  | Sample 11 | Flu B | 24.6 |  |  |  |  |
|  | Sample 24 | RV | 22.6 |  |  |  |  |
|  | Sample 38 | SARS-CoV-2 | 19.7 |  |  |  |  |
| 13 | Sample 30 | RSV | 37.9 | Flu A  RV  RSV  SARS-CoV-2 | 100  100  100  100 | - | - |
|  | Sample 25 | RV | 25.7 |  |  |  |  |
|  | Sample 1 | Flu A | 34.6 |  |  |  |  |
|  | Sample 48 | SARS-CoV-2 | 16.8 |  |  |  |  |
| 14 | Sample 2 | Flu A | 26.5 | Flu A  Flu B  RV  RSV | 100  100  100  100 |  |  |
|  | Sample 10 | Flu B | 32.9 |  |  |  |  |
|  | Sample 20 | RV | 18.2 |  |  |  |  |
|  | Sample 31 | RSV  RV | 27.4  36.3 |  |  |  |  |
| 15 | Sample 6 | Flu A | 39.8 | Flu A  Flu B  NL63  RV  RSV | 100  100  100  100  100 |  |  |
|  | Sample 9 | Flu B  NL63 | 25.8  37.4 |  |  |  |  |
|  | Sample 22 | RV | 20.2 |  |  |  |  |
|  | Sample 28 | RSV | 26.9 |  |  |  |  |
| 16 | Sample 7 | Flu A | 27.6 | Flu A  Flu B  RV  RSV | 100  100  100  100 |  |  |
|  | Sample 11 | Flu B | 24.6 |  |  |  |  |
|  | Sample 50 | RV | 25.7 |  |  |  |  |
|  | Sample 35 | RSV | 29.6 |  |  |  |  |
| 17 | Sample 5 | Flu A | 26.9 | Flu A  Flu B  HKU1  RV  RSV | 100  100  100  100  100 |  |  |
|  | Sample 13 | Flu B  HKU1 | 28.8  37.8 |  |  |  |  |
|  | Sample 51 | RV | 26.9 |  |  |  |  |
|  | Sample 32 | RSV | 39.8 |  |  |  |  |
| 18 | Sample 4 | Flu A | 33 | Flu A  Flu B  RV  RSV | 100  100  100  100 |  |  |
|  | Sample 11 | Flu B | 24.6 |  |  |  |  |
|  | Sample 55 | RV | 31.5 |  |  |  |  |
|  | Sample 26 | RSV | 32 |  |  |  |  |
| 19 | Sample 56 | Flu A | 29.9 | Flu A  Flu B  NL63  RV  RSV | 100  100  100  100  100 |  |  |
|  | Sample 9 | Flu B  NL63 | 25.8  37.4 |  |  |  |  |
|  | Sample 52 | RV | 29.1 |  |  |  |  |
|  | Sample 34 | RSV | 37.6 |  |  |  |  |
| 20 | Sample 1 | Flu A | 34.6 | Flu A  Flu B  RV  RSV | 100  100  100  100 |  |  |
|  | Sample 49 | Flu B | 31.2 |  |  |  |  |
|  | Sample 54 | RV | 26.5 |  |  |  |  |
|  | Sample 30 | RSV | 37.9 |  |  |  |  |
| 21 | Sample 4 | Flu A | 33.0 | Flu A  Flu B  HKU1  RV  RSV | 100  100  100  100  100 |  |  |
|  | Sample 13 | Flu B  HKU1 | 28.8  37.8 |  |  |  |  |
|  | Sample 24 | RV | 22.55 |  |  |  |  |
|  | Sample 33 | RSV | 29.6 |  |  |  |  |
| 22 | Sample 3 | Flu A | 28.8 | Flu A  Flu B  RV  RSV | 100  100  100  100 |  |  |
|  | Sample 49 | Flu B | 31.2 |  |  |  |  |
|  | Sample 53 | RV | 28.6 |  |  |  |  |
|  | Sample 33 | RSV | 28.3 |  |  |  |  |
